# *Mycoplasma bovis* in Spanish Cattle Herds: Two Groups of Multiresistant Isolates Predominate, with One Remaining Susceptible to Fluoroquinolones

**DOI:** 10.3390/pathogens9070545

**Published:** 2020-07-07

**Authors:** Ana García-Galán, Laurent-Xavier Nouvel, Eric Baranowski, Ángel Gómez-Martín, Antonio Sánchez, Christine Citti, Christian de la Fe

**Affiliations:** 1 Ruminant Health Research Group, Department of Animal Health, Faculty of Veterinary Sciences, Regional Campus of International Excellence “*Campus Mare Nostrum*”, University of Murcia, 30100 Murcia, Spain; ana.garcia25@um.es (A.G.-G.); angel.gomezmartin@uchceu.es (A.G.-M.); asanlope@um.es (A.S.); 2 IHAP, ENVT, INRAE, Université de Toulouse, 31300 Toulouse, France; xavier.nouvel@envt.fr (L.-X.N.); eric.baranowski@envt.fr (E.B.); christine.citti@envt.fr (C.C.); 3 Microbiological Agents associated with Reproduction (*ProVaginBio*) Research Group, Department of Animal Health and Public Health, Faculty of Veterinary Sciences, University CEU Cardenal Herrera of Valencia, CEU Universities, 46113 Valencia, Spain

**Keywords:** *Mycoplasma bovis*, minimum inhibitory concentration, antimicrobial resistance, mutations, Spain

## Abstract

*Mycoplasma bovis* is an important bovine pathogen causing pneumonia, mastitis, and arthritis and is responsible for major economic losses worldwide. In the absence of an efficient vaccine, control of *M. bovis* infections mainly relies on antimicrobial treatments, but resistance is reported in an increasing number of countries. To address the situation in Spain, *M. bovis* was searched in 436 samples collected from beef and dairy cattle (2016–2019) and 28% were positive. Single-locus typing using *polC* sequences further revealed that two subtypes ST2 and ST3, circulate in Spain both in beef and dairy cattle, regardless of the regions or the clinical signs. Monitoring of ST2 and ST3 isolates minimum inhibitory concentration (MIC) to a panel of antimicrobials revealed one major difference when using fluoroquinolones (FQL): ST2 is more susceptible than ST3. Accordingly, whole-genome sequencing (WGS) further identified mutations in the *gyrA* and *parC* regions, encoding quinolone resistance-determining regions (QRDR) only in ST3 isolates. This situation shows the capacity of ST3 to accumulate mutations in QRDR and might reflect the selective pressure imposed by the extensive use of these antimicrobials. MIC values and detection of mutations by WGS also showed that most Spanish isolates are resistant to macrolides, lincosamides, and tetracyclines. Valnemulin was the only one effective, at least in vitro, against both STs.

## 1. Introduction

Isolated in the early 60s, *Mycoplasma bovis* is an important bovine pathogen that has a major economic impact on the global cattle industry [1,2]. *M. bovis* is usually associated with a variety of clinical manifestations, including pneumonia, mastitis, arthritis, keratoconjunctivitis, otitis media, and genital disorders [2,3]. In the absence of an efficient vaccine, the control of *M. bovis* infections mainly relies on antimicrobial treatments [4]. However, many countries have reported that the in vitro antimicrobial sensitivity of *M. bovis* isolates has been dramatically reduced [5,6,7,8,9,10,11,12,13,14].

*M. bovis* belongs to the class *Mollicutes*, a large group of wall-less bacteria with reduced genome and limited metabolic capacities, but a remarkable adaptive potential [15,16]. Treatment with ß-lactams, glycopeptides, cycloserines, or fosfomycin is ineffective against *Mollicutes* infections since they all target cell-wall synthesis [17,18]. Similarly, polymyxins and sulfonamides/trimethoprim, whose primary targets are respectively membrane lipopolysaccharides and folic acid, are not effective against these organisms [17,18]. Finally, *Mollicutes* are also resistant to rifampicin due to a natural mutation in the *rpoB* gene of the RNA polymerase β subunit, which prevents the antibiotic from binding to its target [19,20,21]. Antimicrobials active against *Mycoplasmas* include macrolides, lincosamides, tetracyclines, amphenicols, and pleuromutilins, which are all interfering with the synthesis of proteins, and fluoroquinolones (FLQ), which are DNA synthesis inhibitors [22].

General guidelines for antimicrobial testing of veterinary mycoplasmas are available, although no standard or interpretative breakpoint has been formally published [23]. Hence, current minimum inhibitory concentration (MIC) data are supported by molecular evidence of genetic mutations associated with antimicrobial resistance [22,24]. Hot spot mutations in 16S rRNA genes, *rrs3* and *rrs4*, are related to resistance against tetracyclines, while those in 23S rRNA genes, *rrl3* and *rrl4*, are associated with resistance to macrolides, lincosamides, phenicols, and pleuromutilins. Mutations in *rplD* and *rplV* genes encoding ribosomal proteins L4 and L22 and *rplC* gene encoding L3 are also linked to resistance against macrolides and pleuromutilins, respectively. Finally, FLQ resistance is mainly associated with mutations in the quinolone resistance-determining regions (QRDR) of *gyrA* and *gyrB* genes encoding DNA-gyrase, and in *parC* and *parE* genes encoding topoisomerase IV [22,24].

In Europe, *M. bovis* is particularly damaging to the beef industry due to its contribution towards the bovine respiratory disease complex (BRD) that affects calves raised in feedlots [25,26,27]. This pathogen often acts in co-infection with other viruses and bacteria, although it is the only etiological agent found in the chronic forms of the disease [28]. Regarding the dairy industry, sporadic *M. bovis* outbreaks have been notified in Austria, Denmark, Switzerland, and The Netherlands. Based on field data from the analysis of bulk tank milk, the prevalence of the infection in France and the UK is less than 1%, and that in Belgium and Greece it is 1.5% and 5.4%, respectively [29,30,31,32,33,34,35,36].

The beef and dairy industry is crucial to Spain, yet little is known about the epidemiological situation of *M. bovis* infections in this country. The antimicrobial susceptibility of *M. bovis* isolates was recently monitored in different European countries, including Spain [37,38]. However, these studies only considered isolates collected from young animals with respiratory disease and did not provide complete, epidemiological background information regarding the isolates.

The spread of *M. bovis* infection in animals, herds, regions, or countries is usually associated with animal movements and the introduction of asymptomatic carriers, which are occasionally shedding the pathogen in milk, nasal, or genital secretions [2,3]. Animal exchanges between farms are common in the Spanish beef industry, which also imports a large number of animals from other European countries, with France being the main supplier, followed by Ireland and Germany [39]. Animal movements between dairy farms are less common since the replacement of dairy cows is usually performed with animals born in the same herd. Nevertheless, when the replacement rate is not sufficient to maintain milk production levels, external animals may be introduced to the herd, especially in larger farms. Interestingly, a study showed that infected semen was also at the origin of *M. bovis* mastitis outbreaks in two closed dairy herds in Finland [40].

Recently, a large molecular study, including *M. bovis* strains isolated in France from 1977 to 2012, revealed that two groups emerged after 2000 [41]. Based on their partial *polC* sequences, these corresponded to subtypes (STs) 2 and 3. Another study further observed a difference between the two STs in their ability to acquire FLQ resistance in vitro. While ST3 isolates are more likely to acquire mutations in their QRDR and become resistant under selective pressure, the genetic context of ST2 isolates appears to hinder the development of resistance [42]. Field isolates from both STs were found to be resistant to the macrolides tylosin and tilmicosin and the tetracycline, oxytetracycline, regardless of the associated clinical signs (respiratory disease, mastitis, otitis, or arthritis) [43]. Interestingly, the first multiresistant ST3 isolate reported in France was collected in 2011 from a calf born in Spain and raised in a veal-calf herd in Southwest France [41]. This raised the question of how the two STs were distributed in Spanish herds when considering a large number of field isolates, and whether their antimicrobial susceptibility profiles were congruent with *polC* typing. Spain, which allowed unrestricted use of FLQ until very recently, may serve as a clear in vivo model to study the effects of the indiscriminate use of these antimicrobials.

The present study objectives were (i) to assess the circulation of *M. bovis* in Spanish cattle herds using a large collection of isolates collected from beef and dairy cattle and from different sample sources (nasal, auricular, conjunctival, synovial fluid and tissues swabs, and mastitic milk); (ii) to subtype this collection by single-locus sequencing of *polC* [41]; (iii) to determine the antimicrobial susceptibility of *M. bovis* isolates studying differences between STs, with a focus on antimicrobial agents approved to treat bovine respiratory disease and/or mastitis in Spain; and (iv) to assess the occurrence of genetic mutations conferring antimicrobial resistance in a selection of isolates representative of each ST.

## 2. Results

### 2.1. M. bovis Circulating in Spanish Beef and Dairy Herds Belongs to STs 2 and 3

In this study, 93 (35.7%) of the 260 analyzed animals were infected with *M. bovis*. Among the 436 analyzed samples, a total of 165 tested positive for *Mycoplasma* spp. and *M. bovis* was the most commonly found species, with 122 PCR-positive samples.

Among beef cattle, *M. bovis* was detected in 84 (40.9%) of the 205 analyzed animals. Specifically, the pathogen was detected in 81 (44.3%) of the 183 feedlots calves and 3 (13.6%) of the 22 pasture-raised animals. The pathogen was detected in 40 (32%) of the 125 healthy animals and 44 (55%) of the 80 animals with clinical signs of respiratory disease or arthritis. Within the 331 analyzed samples, 102 were tested positive. Most positive samples were obtained from nasal swabs (85/278) and the remaining were identified in auricular swabs (5/27) and tissues swabs from lung (9/16), spleen (1/1), liver (1/2), and mediastinal lymph nodes (1/1). However, the pathogen was not found in conjunctival swabs (n = 3) nor synovial fluid (n = 3). The positive samples were obtained from 26 of the 30 analyzed farms and 5 of the 8 analyzed regions (Figure S1). Among dairy cattle, *M. bovis* was detected in 9 (16.36%) of the 55 analyzed animals. Specifically, the pathogen was detected in 9 (23.1%) of the 39 dairy cows with mastitis but was not detected in any of the 5 dairy calves with clinical signs of respiratory disease nor any of the 11 asymptomatic calves. Within the 105 analyzed samples, positive samples were only detected in mastitic milk (20/66), while any positive results were detected in BTM (n = 9), or nasal (n = 27), auricular (n = 1), or conjunctival (n = 2) swabs. The positive samples were obtained from 2 of the 7 farms and the milk analysis laboratory, and 3 of the 5 analyzed regions (Figure S1).

**Figure 1:**
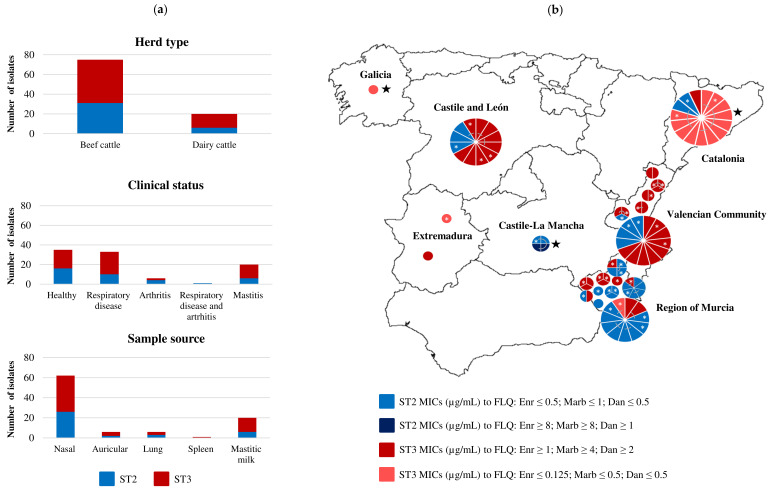
Epidemiological background of the 95 *Mycoplasma bovis* isolates included in this study: (**a**) Number of isolates of each subtype (ST) depending on the herd type, clinical status, and sample source; (**b**) Geographical origin of each isolate. Each circle represents a farm except in Catalonia, where a milk analysis laboratory provided samples. The radius of each circle is proportional to the number of isolates collected from the farm. Isolates collected from mastitic milk are indicated with a black star. Isolates linked with a grey line were obtained from the same animal. Isolates selected for whole-genome sequencing are indicated with a white asterisk. Enr = Enrofloxacin; Marb = Marbofloxacin; Dan = Danofloxacin.

Globally, *M. bovis* was successfully isolated from 112 PCR-positive samples. Based on their origin, 95 representative isolates were chosen for further characterization (epidemiological background provided in Table S1 and illustrated in Figure 1). Briefly, the collection included isolates from beef (n = 75) and dairy cattle (n = 20). Beef cattle isolates were obtained from nasal (62/75), auricular (6/75), lung (6/75) and spleen swabs (1/75), asymptomatic (35/75) or with clinical signs of respiratory disease (33/75), arthritis (6/75), or both (1/75). Dairy cattle isolates were obtained from mastitic milk. Single-locus sequence analysis of *polC* revealed two ST profiles: ST2 (n = 37) and ST3 (n = 58). Both STs were found in beef and dairy cattle, in healthy or diseased animals and in different sample sources. Both STs were found concomitantly in animals from the same farm, or even in different samples from the same animal (Figure 1, Table S1). For example, isolates J96 and J102 (ST3) and J103 (ST2) were collected from spleen, nasal, and lung swabs of the same animal respectively (Table S1). Sequences corresponding to ST2 and ST3 are provided in Table S2.

Hence, no other STs than ST2 or ST3 were found in Spanish herds. Both STs were present in asymptomatic beef cattle or with clinical signs of respiratory disease or arthritis and in dairy cows with mastitis.

### 2.2. The Antimicrobial Susceptibility Profiles of The Spanish Isolates to FLQ Differ Between PolC ST2 and ST3

**Figure 2:**
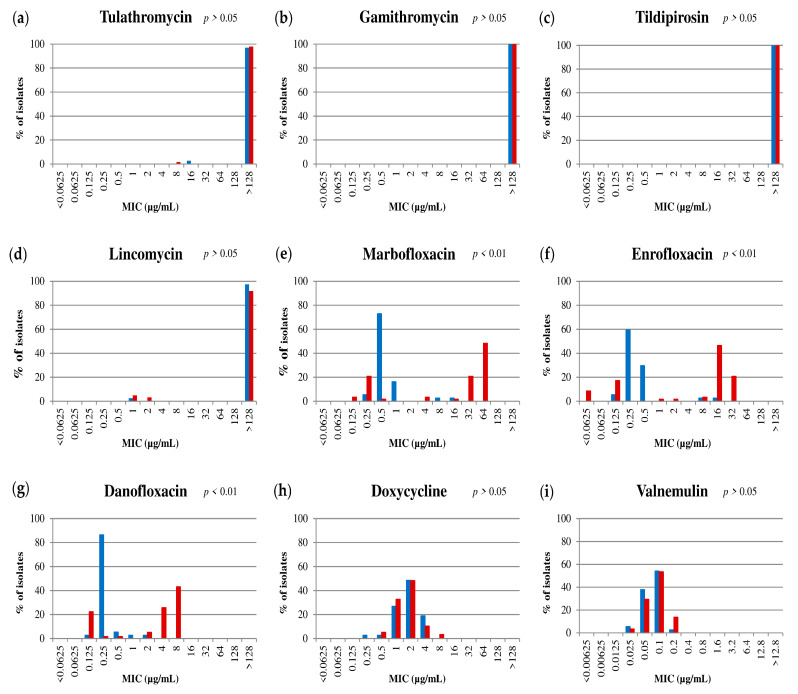
Minimum inhibitory concentration (MIC) distribution (%) of the 95 *Mycoplasma bovis* isolates for each antimicrobial included in this study: (**a**) Tulathromycin; (**b**) Gamithromycin; (**c**) Tildipirosin; (**d**) Lincomycin; (**e**) Marbofloxacin; (**f**) Enrofloxacin; (**g**) Danofloxacin; (**h**) Doxycycline; (**i**) Valnemulin. Blue bars correspond to subtype (ST) 2 and red bars to ST3. *P* values were obtained by comparing the log2MIC means between STs.

The MIC values for the reference strain PG45 are shown in Table 1. Individual MIC values for each isolate are listed in Table S1. Statistical analyses revealed a significant difference in antimicrobial susceptibility to FLQ between ST2 and ST3 isolates (*p* < 0.01). No significant changes between STs were observed for macrolides, lincomycin, doxycycline, or valnemulin. The antimicrobial susceptibility profile of these two STs is illustrated in Table 1, Figure 1 and Figure 2.

MIC values indicated a global decrease of *M. bovis* susceptibility to macrolides and lincomycin (MIC_90_ > 128), and to a lesser extent, doxycycline (MIC_90_ = 4 µg/mL). The majority of ST2 isolates (35/37) had low MIC values for FLQ (≤0.5 µg/mL for enrofloxacin and danofloxacin, and ≤ 1 µg/mL for marbofloxacin) (Figure 1, Table S1). Among the few exceptions were the isolates J320 and J323, obtained from mastitic milk of the same cow. The MIC of J320 was 16 µg/mL for enrofloxacin and marbofloxacin, and 1 µg/mL for danofloxacin and the MIC of J323 was 8 µg/mL for enrofloxacin and marbofloxacin, and 2 µg/mL for danofloxacin (Table S1). Interestingly, 4 ST2 isolates with different MIC profiles were obtained from the cranial quarters of that cow in different days: the isolates J319 (low MIC, left side) and J320 (high MIC, right side) one day, and the isolates J323 (high MIC, left side) and J324 (low MIC, right side) two days later (Figure 1, Table S1). On the contrary, most ST3 (43/58) isolates had high MIC values for FLQ (≥1, ≥4, and ≥2 µg/mL for enrofloxacin, marbofloxacin, and danofloxacin respectively). The remaining ST3 isolates (15/58) were collected from dairy cows with mastitis (13/15) and a few (2/15) from beef cattle with arthritis or asymptomatic (MIC ≤ 0.125 µg/mL for enrofloxacin, and ≤0.5 µg/mL for marbofloxacin and danofloxacin (Figure 1, Table S1). Finally, valnemulin was the only molecule that demonstrated activity against both STs.

Therefore, most of the *M. bovis* Spanish field isolates have a similar antimicrobial susceptibility profile against macrolides, lincomycin, and doxycycline with high MIC values and for valnemulin with low MIC values. On the contrary, antimicrobial susceptibility profiles against FLQ differed between ST2 and ST3, with high MIC values mainly associated with ST3 (Table 1).

### 2.3. Analysis of Point Mutations Conferring Resistance to Antimicrobials: The Main Differences between ST2 and ST3 Are Found in The QRDR of GyrA and ParC Genes

A total of 36 *M. bovis* isolates belonging to ST2 (n = 16) and ST3 (n = 20) were subjected to whole-genome sequencing to compare nucleotide changes at QRDR, and rRNA (16S and 23S) and protein (L3, L4, and L22) genes (Table 2, Table 3 and Table 4). The epidemiological background of these isolates is provided in Table S1 and illustrated in Figure 1.

Nucleotide changes at QRDR revealed important differences between each ST, mainly located in *gyrA* and *parC*. While sequence analysis did not reveal any non-synonymous mutations in *gyrA* or *parC* for ST2 isolates, ST3 isolates were all characterized by at least one non-synonymous mutation in one or both genes. ST3 isolates were all characterized by a *parC* non-synonymous mutation at codon 10 (Gln10Arg). This mutation was associated with a substitution from serine to phenylalanine at *gyrA* codon 83 (Ser83Phe) and serine to isoleucine at *parC* codon 80 (Ser80Ile) in isolates with MIC values ≥ 1 µg/mL for FLQ. Among the few exceptions were the isolates J28, J228, and J279 having no mutation at *parC* codon 80, but a non-synonymous mutation at codon 116 (Ala116Pro in J228 and J279) or codons 81 and 84 (Ser81Pro; Asp84Asn in J28). Interestingly, while most of the ST2 and ST3 isolates showed a *gyrB* non-synonymous mutation associated with a substitution Asp362Asn, ST3 isolates J479, and J482 (MIC values ≥ 8 µg/mL for FLQ) were characterized by a substitution at *gyrB* codon 323 (Val323Ala) in combination with mutations Ser83Phe in *gyrA*, and Gln10Arg, Ser80Ile, and Val156Ile in *parC*.

Mutations in the 23S rRNA and 16S rRNA genes and the ribosomal proteins L3, L4, and L22 are listed in Table 3; Table 4. Regarding 23S rRNA, positions A534T, G748A were notably altered in both *rrl* alleles of all the isolates. Mutation A2058G affecting the majority of isolates (34/36) in one or both alleles was only absent in those with low MIC values for lincomycin (1 µg/mL). Mutations G954A in one or both alleles were altered in 31/36 isolates from both STs and the remaining five isolates had many compensatory non-synonymous mutations in L3, L4, and L22 proteins. Mutation T1249C in one allele was altered in 31/36 isolates from both STs. Mutations A1251T (1/36) and G2157A (5/36) in one allele and G2848T (2/36) in one allele were only found in ST3 isolates while G452A was present in one allele of a few number (5/36) of ST3 isolates. Some isolates from both STs (6/36) showed a single non-synonymous mutation in L4 or L22 (Table 3). Regarding 16S rRNA, mutations A965T and A967T were altered in both *rrs* alleles of all the isolates (MIC ≥ 1 µg/mL for doxycycline). Mutations C1192A in both alleles and T1199C in one or both alleles were altered in 31/36 isolates from both STs. Mutations C335T and C859T were present in one *rrs* allele of five isolates (from both STs) and one isolate (ST2) respectively (Table 4).

Hence, the main differences between ST2 and ST3 are found in the QRDR of *gyrA* and *parC* genes. None of the ST2 isolates have any amino acid substitution in either *gyrA* or *parC* while ST3 isolates with MIC values ≥ 1 µg/mL for FLQ have the mutation Ser83Phe in *gyrA* in combination with at least non-synonymous mutation in *parC* (positions 80, 81, 84, 116, and156).

## 3. Discussion

*M. bovis* was found to be widely distributed in Spanish cattle herds. More specifically, *M. bovis* was mainly detected in feedlot calves (81/183) and to a lesser extent in pasture-raised animals (3/22) housed in 26 different farms from 5 Spanish regions. This pathogenic species was not only detected in animals suffering from respiratory infections and/or arthritis (44/80), but also in asymptomatic carriers (40/125). These results consolidate previous studies that reported the isolation of *M. bovis* from young cattle with respiratory disease in Spain between 2010–2012 and 2015–2016 [37,38]. Although the complete epidemiological background of those isolates was not provided, the authors indicated that each isolate was obtained from a different farm. Altogether, these data indicate that, at least among beef cattle, the infection may have already become endemic, as reported in other European countries [25,26,27]. The presence of asymptomatic carriers and the movement of cattle between beef cattle farms, which frequently involves the mix of animals of diverse origins [39], may explain the current situation in Spain. The isolation of *M. bovis* from clinical mastitis cases was unusual given the low prevalence of this infection in other European countries. Therefore, further studies are needed to confirm whether this particular situation only reflects a bias of the sampling procedure or indicates that Spain is facing an important increase in the number of mastitis cases associated with *M. bovis*.

*M. bovis* isolates circulating in Spain are divided into two *polC* STs, 2 and 3. These two STs are similar to recent French isolates [41,42,43]. Compared with France, where ST2 has been predominant since 2000 [41,42,43], almost two thirds (58/95) of the characterized Spanish isolates belong to ST3. Both STs are widely distributed among different farms and regions, and can be isolated from beef and dairy cattle, from animals with different clinical conditions, and even from different anatomic locations of the same animal. This argues in favor of an efficient circulation and transmission of both STs, as already suggested with French isolates. Thus, animal movement between farms, a common practice in the Spanish beef cattle industry, is likely contributing to the dissemination of *M. bovis* [39]. Animal movements between dairy farms is less common, but asymptomatic carriers can be introduced into the herd when the replacement rate of animals born in the same herd is insufficient to maintain milk production. Furthermore, artificial insemination may be another way of entry for *M. bovis*. This was recently documented in Finland, where semen was reported to be the source of *M. bovis* mastitis outbreaks in two dairy herds [40].

Antimicrobial susceptibility profiles against FLQ differed between ST2 and ST3 isolates. The analysis of the QRDR revealed that the main differences between these STs were located in *gyrA* and *parC*. Remarkably, ST3 isolates were all characterized by an unusual Gln10Arg mutation in *parC*. This mutation is unrelated to antimicrobial resistance, since it was found in ST3 isolates associated with high and low MIC values (≥ 1 and ≤ 1 µg/mL, respectively), and are likely to reflect phylogenetic evolution. ST3 isolates with MIC values ≥ 1 µg/mL were all characterized by mutation Ser83Phe in *gyrA* in combination with one or more amino acid substitution (Ser80Ile, Ser81Pro, Asp84Asn, Ala116Pro, or Val156Ile) in *parC*. Only three of these *parC* mutations, Ser80Ile, Ser81Pro, andAsp84Asn, have been previously described [42,45,46,47,48]. A point mutation Ser83Phe in *GyrA* is sufficient to reach an intermediate level of susceptibility to FLQ but additional substitutions in *parC* are required for resistance [42,44,45,46,47,48]. Interestingly, ST2 and a majority of ST3 (18/20) isolates had the mutation Asp362Asn in *gyrB*. This mutation also appears in recent French isolates and is related to phylogenetic evolution rather than drug resistance [41,42]. Two ST3 isolates harbor a Val323Ala mutation in *gyrB*, but its contribution to FLQ resistance is unknown.

Our results are consistent with in vitro studies showing that under selective pressure, ST3 isolates are more prone to accumulate QRDR mutations than ST2 isolates. Therefore, the widespread circulation of FLQ-resistant ST3 isolates in Spain might reflect the overuse of these antimicrobials in the field. Remarkably, two ST2 isolates were also found to be resistant to FLQ. They were isolated from a cow with clinical mastitis together with susceptible ST2 isolates. This may be the result of long-term treatment with FLQ, leading to the generation of resistant strains, and re-infection with susceptible strains. Globally, our results contrast with other countries where most *M. bovis* strains are susceptible to this family of antimicrobials [6,9,10,11,12,13].

MIC values confirmed the general decrease of *M. bovis* susceptibility to macrolides and lincomycin (MIC_90_ > 128) [5,9,10,11,12,13]. Analysis of 23S rRNA genes revealed that isolates with MIC values > 128 µg/mL for macrolides and lincomycin acquired mutations G748A (in both *rrl* alleles) and A2058G (in one or both *rrl* alleles). A combination of mutations in these hotspots is necessary and sufficient to achieve resistance to other macrolides, such as tylosin and tilmicosin, while mutation A2058G in one or both alleles has been linked to lincomycin resistance in *M. synoviae* [43,49,50]. Isolates J28 and J137 showed high MIC values (16–128 µg/mL) for macrolides but did not carry the mutation A2058G. Consistently, they are the only isolates with low MIC values for lincomycin (1 µg/mL). However, both isolates have several non-synonymous mutations in L4 and L22 proteins including Gln93His in L22, which is related to macrolide resistance and could explain the observed high MIC values for these antimicrobials [43]. No other point mutations related to antimicrobial resistance have been found in the *rrl* alleles or in L4 and L22 proteins. Since they appear together with other mutations conferring resistance, it is difficult to determine their importance.

As expected by the in vitro antimicrobial activity of pleuromutilins against a broad range of veterinary mycoplasmas [22], valnemulin was the only antimicrobial that demonstrated activity against both STs. Indeed, no mutation previously associated with pleuromutilin resistance [47] has been observed in any isolate. This is consistent with the fact that pleuromutilins are only registered for treatment in swine and poultry [52]. Valnemulin may thus be an interesting therapeutic alternative as it has been shown to be effective for the treatment of calves experimentally infected with *M. bovis* [53].

Overall, low in vitro susceptibility was observed for doxycycline (MIC_90_ = 4 µg/mL). Analysis of 16S rRNA genes revealed that isolates with MIC values ≥ 1 µg/mL were characterized by mutations A965T and A967T in both *rrs* alleles. Previous studies have concluded that this double mutation causes decreased susceptibility to other antimicrobials from the same group, such as oxytetracycline and tetracycline [43,51]. Mutations C1192A and T1199C were previously described in French isolates [43], although they did not further modify MIC values as it occurs with Spanish isolates. However, the mutation C1192A has been described both in Hungarian and Japanese isolates and was associated with high MIC values for spectinomycin [47,48]. As expected, mutations C335T and C859T, which have never been associated with antimicrobial resistance, had no influence on the susceptibility of the Spanish isolates. Finally, our results were also consistent with data suggesting that after macrolides, the highest resistances of the main veterinary mycoplasmas species are observed for tetracyclines [22].

In conclusion, our study revealed the extended circulation of *M. bovis* in Spanish beef cattle herds and its implication in mastitis cases. Circulating isolates are divided into two groups, ST2 and ST3, both being resistant to macrolides, lincosamides and tetracyclines. Most ST3 isolates circulating in Spain are resistant to FLQ, a situation which illustrates the remarkable capacity of ST3 to accumulate mutations in QRDR and the selective pressure imposed by the indiscriminate use of these antimicrobials. Valnemulin has been shown to be very effective against both STs in vitro, and its effectiveness in vivo should be further investigated.

## 4. Materials and Methods

### 4.1. Animal Sampling

All animal procedures were performed following the EU Directive 2010/63/EU for animal experimentation and had the authorization of the Ethics Committee on Animal Testing of the University of Murcia (Number: 307/2017).

In this study, 260 animals from 10 Spanish regions were sampled over a 4 year period (2016–2019). A total of 433 samples were collected from beef and dairy cattle.

Among beef cattle, 183 calves were raised in feedlots and 22 pasture-raised animals were sampled. Healthy animals (n = 125) and animals with clinical symptoms of respiratory disease or arthritis (n = 80) were both considered. In total, 331 samples were obtained from beef cattle. The sample collection was composed of nasal swabs (n = 278), auricular (n = 27) and conjunctival swabs (n = 3), synovial fluid (n = 3), as well as a number of swabs from tissues (lung, n = 16; liver, n = 2; spleen, n = 1; and mediastinal lymph node, n = 1). Those samples were obtained from 30 farms and 8 different regions (Figure S1).

Among dairy cattle, 39 cows with mastitis, and 16 calves with clinical signs of respiratory disease (n = 5) or asymptomatic (n = 11) were sampled. In total, 105 samples were obtained from dairy cattle. The sample collection was composed of mastitic milk (n = 66), bulk tank milk (BTM) (n = 9), and nasal (n = 27), auricular (n = 1), and conjunctival swabs (n = 2). Those samples were obtained from 7 farms and a milk analyses laboratory that provided samples and they were taken from 5 different regions (Figure S1).

### 4.2. Mycoplasma Isolation and Subtyping

For mycoplasma isolation from animal samples, swabs or mastitic milk samples (200 µL) were incubated at 37 °C for 24 h in 2 mL of SP4 medium [54] with modifications (Appendix A). Cultures were filtered through a 0.45 μm membrane filter (LLG-Labware, UK) and further incubated for 48 h before plating 5 µL onto solid SP4 medium. Agar plates were grown at 37 °C and examined daily under the microscope for the presence of mycoplasma colonies with the typical fried egg morphology.

The DNA extraction was performed from 200 µL of culture [55]. *M. bovis* detection was performed by PCR amplification of the membrane protein 81 gene [56]. *M. bovis* PCR positive cultures were three times cloned by picking single colonies and the identity of the final isolate was confirmed again by PCR.

*M. bovis* subtyping was performed by sequence analysis of a 520 bp region of the *polC* gene, as previously described [41]. Amplicon sequencing was performed at the molecular biology service of the University of Murcia and sequence analyses were conducted using MEGA 6 [57].

### 4.3. MIC Assays

Antimicrobials used for MIC assays included (i) the macrolides, tulathromycin (Carbosiynth, Compton, UK), gamithromycin (Sigma-Aldrich, St. Louis, MO, USA) and tildipirosin (Carbosiynth, Compton, UK), (ii) the lincosamide, lincomycin (Sigma-Aldrich, St. Louis, MO, USA), (iii) the FLQ, enrofloxacin (Fluka, Bio-Chemika, St. Louis, MO, USA), marbofloxacin (Tokio Chemical Industry, Chuo City, Japan) and danofloxacin (Fluka, Bio-Chemika, St. Louis, MO, USA), (iv) the tetracycline, doxycycline (Sigma-Aldrich, St. Louis, MO, USA), and (v) the pleuromutilin, valnemulin hydrochloride (Sigma-Aldrich, St. Louis, MO, USA). Stock solutions (1 mg/mL; 0.1 mg/mL for valnemulin hydrochloride) and two-fold dilutions were prepared in sterile distilled water. For preparing enrofloxacin, marbofloxacin, and danofloxacin, 0.1 M HCl was added dropwise until dissolution occurred and the volume was adjusted with sterile distilled water. A final range from 128 µg/mL to 0.0625 µg/mL was tested except for valnemulin, for which a final range from 12.8 µg/mL to 0.00625 µg/mL was studied.

Stationary-phase cultures of 95 *M. bovis* isolates and the reference strain PG45 were used for MIC assays. Mycoplasma cultures were carried out in PH medium [58] without antimicrobials, supplemented with sodium pyruvate (0.5%) and phenol red (0.005%), and mycoplasma titers were determined as previously described [59]. MIC assays were carried out in 96-well microtiter plates using the microbroth dilution method [23]. Briefly, 25.6 µL of each antimicrobial dilution and 25 µL of the diluted *M. bovis* inoculum (10^3^–10^5^ CFU/mL) were added to 150 µL of culture medium. Additionally, a positive control (well without antimicrobial) and a negative control (well without neither antimicrobial nor inoculum) were included in each essay. After 48 h of incubation at 37 °C, plates were examined for color change. MIC was defined as the lowest concentration of antimicrobial capable of completely inhibiting the growth of *M. bovis*. For each antimicrobial, the MIC range, MIC_50_ (lowest concentration of antimicrobial capable of inhibiting the growth of 50% of the isolates), and MIC_90_ (lowest concentration of antimicrobial capable of inhibiting the growth of 90% of the isolates) were calculated. All the assays were performed in duplicate. For accepting the results, MIC values of the duplicate tests had to be within one dilution, with the higher MIC value being used. If not, a third assay was performed, and the final MIC value was the mode of the three values.

### 4.4. Statistical Analysis

MIC values were transformed to a continuous variable by calculating their Log2 values. Log2MIC means values of ST2 and ST3 isolates were compared for each antimicrobial. Statistical analyses were run using the EpiInfo software [60] using ANOVA or Mann–Whitney/Wilcoxon Two-Sample Test (Kruskal–Wallis test for two groups) according to the inequality of population variances and with the significance level set at 0.01.

### 4.5. Whole-Genome Sequencing

Genomic DNA was extracted from a selection of 36 isolates (Table S1) from 15 mL of mycoplasma culture using a High Pure PCR Template Preparation Kit (Roche, Bâle, Suisse) according to the manufacturer’s instructions. Whole-genome sequencing was performed using Illumina technology Hiseq (paired-end, 2 × 150pb) by Novogene Europe (Cambridge, UK). Bioinformatics analyses were performed on Galaxy platform (Genotoul, Toulouse, France). Quality controls of reads were performed using *FastQC* tool [61]. Alignments were carried out with *BWA-MEM* using PG45 as the reference [62], and alignments quality controls were checked with *QualiMap BamQC* [63]. SNP identification was done by alignment visualization with *Integrative Genomics Viewer* (IGV 2.7.0) [64] or by variant calling analysis with *breseq* [65]. All sequence files are available from the European Nucleotide Archive database (ENA), under study accession number PRJEB38707.

## Figures and Tables

**Table 1:** Minimum inhibitory concentration (MIC) ranges, MIC_50_ and MIC_90_ of *Mycoplasma bovis* isolates.

*polC*^a^ ST	MIC Parameter	Macrolides			Lincosamide	Fluoroquinolones			Tetracycline	Pleuromutilin
		Tul	Gam	Tild	Lin	Enr	Marb	Dan	Dox	Val
1 PG45	MIC	1	8	1	1	0.125	0.5	0.125	0.0625	0.025
2 (n = 37)	MIC Range	16–>128	>128	>128	1–>128	0.125–16	0.25–16	0.125–2	0.25–4	0.025–0.2
	MIC_50_	>128	>128	>128	>128	0.25	0.5	0.25	2	0.1
	MIC_90_	>128	>128	>128	>128	0.5	1	0.5	4	0.1
3 (n = 58)	MIC Range	8–>128	>128	>128	1–>128	<0.0625–32	0.125–64	0.125–8	0.5–8	0.025–0.2
	MIC_50_	>128	>128	>128	>128	16	32	4	2	0.1
	MIC_90_	>128	>128	>128	>128	32	64	8	4	0.2

MIC values are given in µg/mL. Values are presented separately for each subtype (ST). The reference strain PG45 was used as control. Tul = Tulathromycin; Gam = Gamithromycin; Tild = Tildipirosin; Lin = Lincomycin; Enr = Enrofloxacin; Marb = Marbofloxacin; Dan = Danofloxacin; Dox = Doxycycline; Val = Valnemulin. ^a^ ST based on the single-locus sequence analysis of a region of the gene *polC* [41].

**Table 2:** List of point mutations in the *gyrA*, *gyrB*, and *parC* quinolone resistance-determining regions (QRDR) identified in *Mycoplasma bovis* isolates and associated minimum inhibitory concentration (MIC) values for fluoroquinolones (FLQ).

Isolate	*polC*^a^ ST	*gyrA*	*gyrB*		*parC*						MIC (µg/mL) ^b^		
		83 ^c^	362	323	10	80 ^c^	81 ^c^	84 ^c^	116	156	Enr	Marb	Dan
PG45	1	Ser	Asp	Val	Gln	Ser	Ser	Asp	Ala	Val	0.125	0.5	0.125
J335	3	-	Asn	-	Arg	-	-	-	-	-	<0.0625	0.25	0.125
J403	3	-	Asn	-	Arg	-	-	-	-	-	<0.0625	0.25	0.125
J414	3	-	Asn	-	Arg	-	-	-	-	-	<0.0625	0.25	0.125
J433	3	-	Asn	-	Arg	-	-	-	-	-	0.125	0.25	0.125
J341	2	-	Asn	-	-	-	-	-	-	-	0.125	0.25	0.25
J6	2	-	Asn	-	-	-	-	-	-	-	0.25	0.5	0.25
J103	2	-	Asn	-	-	-	-	-	-	-	0.25	0.5	0.25
J175	2	-	Asn	-	-	-	-	-	-	-	0.25	0.5	0.25
J226	2	-	Asn	-	-	-	-	-	-	-	0.25	0.5	0.25
J276	2	-	Asn	-	-	-	-	-	-	-	0.25	0.5	0.25
J319	2	-	Asn	-	-	-	-	-	-	-	0.25	0.5	0.25
J330	2	-	Asn	-	-	-	-	-	-	-	0.25	0.5	0.25
J336	2	-	Asn	-	-	-	-	-	-	-	0.25	0.5	0.5
J356	2	-	Asn	-	-	-	-	-	-	-	0.25	0.5	0.25
J136	2	-	Asn	-	-	-	-	-	-	-	0.5	1	0.25
J137	2	-	Asn	-	-	-	-	-	-	-	0.5	0.5	0.125
J368	2	-	Asn	-	-	-	-	-	-	-	0.5	1	0.25
J377	2	-	Asn	-	-	-	-	-	-	-	0.5	1	0.25
J391	2	-	Asn	-	-	-	-	-	-	-	0.5	0.5	0.25
J410	2	-	Asn	-	-	-	-	-	-	-	0.5	0.5	0.25
J279	3	Phe	Asn	-	Arg	-	-	-	Pro	-	1	4	4
J228	3	Phe	Asn	-	Arg	-	-	-	Pro	-	2	4	2
J115	3	Phe	Asn	-	Arg	Ile	-	-	-	-	8	32	2
J28	3	Phe	Asn	-	Arg	-	Pro	Asn	-	-	16	64	8
J69	3	Phe	Asn	-	Arg	Ile	-	-	-	-	16	32	4
J72	3	Phe	Asn	-	Arg	Ile	-	-	-	-	16	64	8
J81	3	Phe	Asn	-	Arg	Ile	-	-	-	-	16	32	4
J96	3	Phe	Asn	-	Arg	Ile	-	-	-	-	16	32	4
J131	3	Phe	Asn	-	Arg	Ile	-	-	-	-	16	64	8
J305	3	Phe	Asn	-	Arg	Ile	-	-	-	-	16	64	8
J178	3	Phe	Asn	-	Arg	Ile	-	-	-	-	32	64	8
J233	3	Phe	Asn	-	Arg	Ile	-	-	-	-	32	64	8
J295	3	Phe	Asn	-	Arg	Ile	-	-	-	-	32	64	8
J388	3	Phe	Asn	-	Arg	Ile	-	-	-	-	32	64	8
J479	3	Phe	-	Ala	Arg	Ile	-	-	-	Ile	32	64	8
J482	3	Phe	-	Ala	Arg	Ile	-	-	-	Ile	32	64	8

Amino acid numbering refers to positions in *Escherichia coli* K12. ^a^ Subtype (ST) based on the single-locus sequence analysis of a region of the gene *polC* [41]. ^b^ Enr = Enrofloxacin; Marb = Marbofloxacin; Dan = Danofloxacin. **^c^** Mutations associated with FLQ resistance in previous studies [42,44,45,46,47,48].

**Table 3:** List of point mutations in 23S rRNA alleles of *Mycoplasma bovis* isolates and associated minimum inhibitory concentration (MIC) values for macrolides, lincomycin, and valnemulin.

Isolate	*polC*^a^ ST	23S rRNA, *rrl* alleles ^b^									L3 ^c^	L4 ^c^												L22 ^c^		MIC (µg/mL) ^d^				
		452	534	748 ^e^	954	1249	1251	2058 ^e,f^	2157	2848	265	11	24	36	44	62	63	68	79	94	178	178	178	5	93 ^e^	Tul	Gam	Tild	Lin	Val
PG45	1	G	A	G	G	T	A	A	G	G	Ala	Ser	Thr	Thr	Ala	Val	Ala	Glu	Ala	Ala	Gly	Gly	Gly	Gln	Gln	1	8	1	1	0.025
J137	2	-	T **	A **	-	C *	-	-	-	-	Val	Thr	-	Ala	Thr	Ala	Thr	Ala	Thr	Thr	-	-	Val	-	His	16	>128	>128	1	0.1
J28	3	-	T **	A **	-	C *	-	-	-	-	Val	Thr	-	Ala	Thr	Ala	Thr	Ala	Thr	Thr	-	Leu	-	-	His	>128	>128	>128	1	0.05
J403	3	-	T **	A **	-	C *	-	G *	-	-	Val	Thr	-	-	Thr	Ala	Thr	Ala	Thr	Thr	Arg	-	-	-	His	>128	>128	>128	>128	0.1
J414	3	-	T **	A **	-	C *	-	G *	-	-	Val	Thr	-	Ala	Thr	Ala	Thr	Ala	Thr	Thr	Arg	-	-	-	His	>128	>128	>128	>128	0.1
J433	3	-	T **	A **	-	C *	-	G *	-	-	Val	Thr	-	Ala	Thr	Ala	Thr	Ala	Thr	Thr	Arg	-	-	-	His	>128	>128	>128	>128	0.1
J6	2	-	T **	A **	A *	C *	-	G **	-	-	-	-	-	-	-	-	-	-	-	-	-	-	-	-	-	>128	>128	>128	>128	0.1
J103	2	-	T **	A **	A *	C *	-	G **	-	-	-	-	Arg	-	-	-	-	-	-	-	-	-	-	-	-	>128	>128	>128	>128	0.025
J136	2	-	T **	A **	A *	C *	-	G **	-	-	-	-	-	-	-	-	-	-	-	-	-	-	-	Lys	-	>128	>128	>128	>128	0.05
J175	2	-	T **	A **	A *	C *	-	G **	-	-	-	-	-	-	-	-	-	-	-	-	-	-	-	-	-	>128	>128	>128	>128	0.05
J226	2	-	T **	A **	A *	C *	-	G **	-	-	-	-	-	-	-	-	-	-	-	-	-	-	-	-	-	>128	>128	>128	>128	0.1
J276	2	-	T **	A **	A *	C *	-	G **	-	-	-	-	-	-	-	-	-	-	-	-	-	-	-	-	-	>128	>128	>128	>128	0.05
J319	2	-	T **	A **	A *	C *	-	G **	-	-	-	-	-	-	-	-	-	-	-	-	-	-	-	-	-	>128	>128	>128	>128	0.05
J330	2	-	T **	A **	A *	C *	-	G **	-	-	-	-	-	-	-	-	-	-	-	-	-	-	-	-	-	>128	>128	>128	>128	0.1
J336	2	-	T **	A **	A *	C *	-	G **	-	-	-	-	-	-	-	-	-	-	-	-	-	-	-	-	-	>128	>128	>128	>128	0.05
J341	2	-	T **	A **	A *	C *	-	G **	-	-	-	-	-	-	-	-	-	-	-	-	-	-	-	-	-	>128	>128	>128	>128	0.05
J356	2	-	T **	A **	A *	C *	-	G **	-	-	-	-	-	-	-	-	-	-	-	-	-	-	-	-	-	>128	>128	>128	>128	0.1
J368	2	-	T **	A **	A *	C *	-	G **	-	-	-	-	-	-	-	-	-	-	-	-	-	-	-	-	-	>128	>128	>128	>128	0.1
J377	2	-	T **	A **	A *	C *	-	G **	-	-	-	-	-	-	-	-	-	-	-	-	-	-	-	-	-	>128	>128	>128	>128	0.05
J391	2	-	T **	A **	A *	C *	-	G **	-	-	-	-	-	-	-	-	-	-	-	-	-	-	-	-	-	>128	>128	>128	>128	0.2
J410	2	-	T **	A **	A *	C *	-	G **	-	-	-	-	-	-	-	-	-	-	-	-	-	-	-	-	-	>128	>128	>128	>128	0.1
J96	3	-	T **	A **	A *	C *	-	G **	A *	-	-	-	-	-	-	-	-	-	-	-	-	-	-	-	-	>128	>128	>128	>128	0.1
J178	3	-	T **	A **	A *	C *	-	G **	-	-	-	-	-	-	-	-	-	-	-	-	-	-	-	-	-	>128	>128	>128	>128	0.05
J228	3	-	T **	A **	A *	C *	-	G **	-	-	-	-	-	-	Thr	-	-	-	-	-	-	-	-	-	-	>128	>128	>128	>128	0.1
J233	3	-	T **	A **	A *	C *	-	G **	A *	-	-	-	-	-	-	-	-	-	-	-	-	-	-	-	-	>128	>128	>128	>128	0.1
J279	3	-	T **	A **	A *	C *	-	G **	-	-	-	-	-	-	-	-	-	-	-	-	-	-	-	-	-	>128	>128	>128	>128	0.1
J295	3	-	T **	A **	A *	C *	-	G **	A *	-	-	-	-	-	-	-	-	-	-	-	-	-	-	-	-	>128	>128	>128	>128	0.1
J305	3	-	T **	A **	A *	C *	-	G **	A *	-	-	-	-	-	-	-	-	-	-	-	-	-	-	-	-	>128	>128	>128	>128	0.1
J335	3	-	T **	A **	A *	C *	T*	G **	-	-	-	-	-	-	-	-	-	-	-	-	-	-	-	-	-	>128	>128	>128	>128	0.05
J388	3	-	T **	A **	A *	C *	-	G **	A *	-	-	-	-	-	-	-	-	-	-	-	-	-	-	-	-	>128	>128	>128	>128	0.1
J479	3	-	T **	A **	A *	C *	-	G **	-	-	-	-	-	-	-	-	-	-	-	-	-	-	-	-	-	>128	>128	>128	>128	0.2
J482	3	-	T **	A **	A *	C *	-	G **	-	-	-	-	-	-	-	-	-	-	-	-	-	-	-	-	-	>128	>128	>128	>128	0.2
J69	3	A *	T **	A **	A **	-	-	G **	-	-	-	-	-	-	Thr	-	-	-	-	-	-	-	-	-	-	>128	>128	>128	>128	0.05
J72	3	A *	T **	A **	A **	-	-	G **	-	-	-	-	-	-	-	-	-	-	-	-	-	-	-	-	-	>128	>128	>128	>128	0.05
J81	3	A *	T **	A **	A **	-	-	G **	-	T *	-	-	-	-	-	-	-	-	-	-	-	-	-	-	-	>128	>128	>128	>128	0.1
J115	3	A *	T **	A **	A **	-	-	G **	-	T *	-	-	-	-	Thr	-	-	-	-	-	-	-	-	-	-	>128	>128	>128	>128	0.025
J131	3	A *	T **	A **	A **	-	-	G **	-	-	-	-	-	-	Thr	-	-	-	-	-	-	-	-	-	-	>128	>128	>128	>128	0.1

^a^ Subtype (ST) based on the single-locus sequence analysis of a region of the gene *polC* [41]; ^b^ nucleotide numbering refers to *Escherichia coli* K12; a single * indicates mutation in one *rrl* allele and ** indicates mutation in both alleles; ^c^ amino acid numbering refers to positions in PG45; ^d^ Tul= Tulathromycin; Gam = Gamithromycin; Tild = Tildipirosin; Lin = Lincomycin; Val = Valnemulin. **^e^** Mutations associated with macrolides resistance in *M. bovis* [43,47,49]. **^f^** Mutation associated with lincomycin resistance in *Mycoplasma synoviae* [50].

**Table 4:** List of point mutations in 16S rRNA alleles of *Mycoplasma bovis* isolates and associated minimum inhibitory concentration (MIC) values for doxycycline.

Isolate	*polC*^a^ ST	16S rRNA, *rrs* alleles ^b^						MIC (µg/mL) ^c^
		335	859	965 ^d^	967 ^d^	1192 ^e^	1199	Dox
PG45	1	C	C	A	A	C	T	0.0625
J137	2	T *	T *	T **	T **	-	-	1
J28	3	T *	-	T **	T **	-	-	1
J403	3	T *	-	T **	T **	-	-	1
J414	3	T *	-	T **	T **	-	-	1
J433	3	T *	-	T **	T **	-	-	1
J276	2	-	-	T **	T **	A **	C **	1
J319	2	-	-	T **	T **	A **	C **	1
J341	2	-	-	T **	T **	A **	C **	1
J115	3	-	-	T **	T **	A **	C **	1
J335	3	-	-	T **	T **	A **	C **	1
J6	2	-	-	T **	T **	A **	C **	2
J103	2	-	-	T **	T **	A **	C **	2
J136	2	-	-	T **	T **	A **	C **	2
J175	2	-	-	T **	T **	A **	C **	2
J226	2	-	-	T **	T **	A **	C **	2
J336	2	-	-	T **	T **	A **	C **	2
J356	2	-	-	T **	T **	A **	C **	2
J377	2	-	-	T **	T **	A **	C **	2
J391	2	-	-	T **	T **	A **	C **	2
J410	2	-	-	T **	T **	A **	C **	2
J69	3	-	-	T **	T **	A **	C **	2
J72	3	-	-	T **	T **	A **	C **	2
J81	3	-	-	T **	T **	A **	C **	2
J178	3	-	-	T **	T **	A **	C **	2
J228	3	-	-	T **	T **	A **	C *	2
J279	3	-	-	T **	T **	A **	C *	2
J295	3	-	-	T **	T **	A **	C **	2
J305	3	-	-	T **	T **	A **	C **	2
J479	3	-	-	T **	T **	A **	C *	2
J482	3	-	-	T **	T **	A **	C *	2
J330	2	-	-	T **	T **	A **	C **	4
J368	2	-	-	T **	T **	A **	C **	4
J131	3	-	-	T **	T **	A **	C **	4
J233	3	-	-	T **	T **	A **	C **	4
J96	3	-	-	T **	T **	A **	C **	8
J388	3	-	-	T **	T **	A **	C **	8

^a^ Subtype (ST) based on the single-locus sequence analysis of a region of the gene *polC* [41]; ^b^ nucleotide numbering refers to *Escherichia coli K12*; a single * indicates mutation in one *rrl* allele and ** indicates mutation in both alleles; ^c^ Dox = doxycycline. ^d^ Mutations associated with *M bovis* tetracyclines resistance in previous studies [43,51]. ^e^ Mutation associated with spectinomycin resistance in previous studies [47,48].

## Supplementary Materials

The following are available online at https://www.mdpi.com/2076-0817/9/7/545/s1, Figure S1: Map of Spain showing the autonomous communities (AC) and the origin of the samples, Table S1: Epidemiological background, *polC* characterization and minimum inhibitory concentration (MIC) values of the 95 *Mycoplasma bovis* isolates, Table S2: Partial sequences (520 pb) types of the *polC* gene.

## Appendix A

The medium SP4 was prepared following previous recommendations [54] but with some modifications.The modified medium is composed of three parts (A, B, and C). Part A is composed of 4.2 g of Difco PPLO broth (BD), 6.4 g of Bacto Peptone (BD), 12 g of Bacto Tryptone (BD) and 724 mL of deionized water. The solid medium includes 7 g of European Bacteriological Agar (Conda-Pronadisa). The pH is adjusted to 7.8 and then part A is autoclaved at 121 °C for 20 min. Part B is composed of 60 mL of RPMI-1640 (Sigma-Aldrich), 21 mL of fresh yeast extract 50% *w*/*v*, 2.4 g of yeast extract (Conda-Pronadisa), 4.8 mL of phenol red 0.5%, (Sigma-Aldrich) and 0.642 g of ampicillin sodium salt (Fisher bioreagents). The pH is adjusted to 7.2 and then part B is filter-sterilized through a 0.2 µL pore size filter. Part C is composed of 251 mL of heat-inactivated horse serum (Hyclone) for 30 min at 56 °C.

## References

[1] Hale H.H., Helmboldt C.F., Plastridge W.N., Stula E.F. (1962). Bovine mastitis caused by a Mycoplasma species. Cornell Vet..

[2] Nicholas R.A.J., Ayling R.D. (2003). *Mycoplasma bovis*: Disease, diagnosis, and control. Res. Vet. Sci..

[3] Maunsell F.P., Woolums A.R., Francoz D., Rosenbusch R.F., Step D.L., Wilson D.J., Janzen E.D. (2011). *Mycoplasma bovis* infections in cattle. J. Vet. Intern. Med..

[4] Perez-Casal J., Prysliak T., Maina T., Suleman M., Jimbo S. (2017). Status of the development of a vaccine against Mycoplasma bovis. Vaccine.

[5] Gerchman I., Levisohn S., Mikula I., Lysnyansky I. (2009). In vitro antimicrobial susceptibility of *Mycoplasma bovis* isolated in Israel from local and imported cattle. Vet. Microbiol..

[6] Soehnlen M.K., Kunze M.E., Karunathilake K.E., Henwood B.M., Kariyawasam S., Wolfgang D.R., Jayarao B.M. (2011). In vitro antimicrobial inhibition of *Mycoplasma bovis* isolates submitted to the Pennsylvania Animal Diagnostic Laboratory using flow cytometry and a broth microdilution method. J. Vet. Diagn..

[7] Hendrick S.H., Bateman K.G., Rosengren L.B. (2013). The effect of antimicrobial treatment and preventive strategies on bovine respiratory disease and genetic relatedness and antimicrobial resistance of *Mycoplasma bovis* isolates in a western Canadian feedlot. Can. Vet. J. Rev. Vet. Can..

[8] Ayling R.D., Rosales R.S., Barden G., Gosney F.L. (2014). Changes in antimicrobial susceptibility of *Mycoplasma bovis* isolates from Great Britain. Vet. Rec..

[9] Gautier-Bouchardon A.V., Ferré S., Le Grand D., Paoli A., Gay E., Poumarat F. (2014). Overall decrease in the susceptibility of *Mycoplasma bovis* to antimicrobials over the past 30 years in France. PLoS ONE.

[10] Kawai K., Higuchi H., Iwano H., Iwakuma A., Onda K., Sato R., Hayashi T., Nagahata H., Oshida T. (2014). Antimicrobial susceptibilities of Mycoplasma isolated from bovine mastitis in Japan. Anim. Sci. J..

[11] Sulyok K.M., Kreizinger Z., Fekete L., Hrivnák V., Magyar T., Jánosi S., Schweitzer N., Turcsányi I., Makrai L., Erdélyi K. (2014). Antibiotic susceptibility profiles of *Mycoplasma bovis* strains isolated from cattle in Hungary, Central Europe. BMC Vet. Res..

[12] Heuvelink A., Reugebrink C., Mars J. (2016). Antimicrobial susceptibility of *Mycoplasma bovis* isolates from veal calves and dairy cattle in the Netherlands. Vet. Microbiol..

[13] Kong L.C., Gao D., Jia B.Y., Wang Z., Gao Y.-H., Pei Z.-H., Liu S.-M., Xin J.-Q., Ma H.-X. (2016). Antimicrobial susceptibility and molecular characterization of macrolide resistance of *Mycoplasma bovis* isolates from multiple provinces in China. J. Vet. Med. Sci..

[14] Jelinski M., Kinnear A., Gesy K., Andrés-Lasheras S., Zaheer R., Weese S., McAllister T.A. (2020). Antimicrobial sensitivity testing of *Mycoplasma bovis* isolates derived from Western Canadian feedlot cattle. Microorganisms.

[15] Citti C., Dordet-Frisoni E., Nouvel L.X., Kuo C., Baranowski E. (2018). Horizontal gene transfers in mycoplasmas (Mollicutes). Curr. Issues Mol. Biol..

[16] Faucher M., Nouvel L.-X., Dordet-Frisoni E., Sagné E., Baranowski E., Hygonenq M.-C., Marenda M.-S., Tardy F., Citti C. (2019). Mycoplasmas under experimental antimicrobial selection: The unpredicted contribution of horizontal chromosomal transfer. PLoS Genet..

[17] McCormack W.M. (1993). Susceptibility of mycoplasmas to antimicrobial agents: Clinical implications. Clin. Infect. Dis..

[18] Olaitan A.O., Morand S., Rolain J.M. (2014). Mechanisms of polymyxin resistance: Acquired and intrinsic resistance in bacteria. Front. Microbiol..

[19] Gadeau A.P., Mouches C., Bove J.M. (1986). Probable insensitivity of mollicutes to rifampin and characterization of spiroplasmal DNA-dependent RNA polymerase. J. Bacteriol..

[20] Pellegrin J.L., Maugein J., Clerc M.T., Leng B., Bové J.M., Bébéar C. (1990). Activity of rifampin against Mollicutes, clostridia and L forms. Recent advances in mycoplasmology. Zentralbl. Bakteriol. Suppl..

[21] Shepard M.C., Lunceford C.D., Ford D.K., Purcell R.H., Taylor-Robinson D., Razin S., Black F.T. (1974). Ureaplasma urealyticum gen. nov., sp. nov.: Proposed nomenclature for the human T (T-Strain) mycoplasmas. Int. J. Syst. Evol. Bacteriol..

[22] Gautier-Bouchardon A.V. (2018). Antimicrobial resistance in mycoplasma spp.. Microbiol. Spectr..

[23] Hannan P.C. (2000). Guidelines and recommendations for antimicrobial minimum inhibitory concentration (MIC) testing against veterinary mycoplasma species. International research programme on comparative mycoplasmology. Vet. Res..

[24] Lysnyansky I., Ayling R.D. (2016). Mycoplasma bovis: Mechanisms of resistance and trends in antimicrobial susceptibility. Front. Microbiol..

[25] Arcangioli M.-A., Duet A., Meyer G., Dernburg A., Bézille P., Poumarat F., Le Grand D. (2008). The role of *Mycoplasma bovis* in bovine respiratory disease outbreaks in veal calf feedlots. Vet. J..

[26] Nicholas R.A.J. (2011). Bovine mycoplasmosis: Silent and deadly. Vet. Rec..

[27] Radaelli E., Luini M., Loria G.R., Nicholas R.A.J., Scanziani E. (2008). Bacteriological, serological, pathological and immunohistochemical studies of *Mycoplasma bovis* respiratory infection in veal calves and adult cattle at slaughter. Res. Vet. Sci..

[28] Caswell J.L., Bateman K.G., Cai H.Y., Castillo-Alcala F. (2010). *Mycoplasma bovis* in respiratory disease of feedlot cattle. Vet. Clin. North Am. Food Anim. Pract..

[29] Nielsen P.K., Petersen M.B., Nielsen L.R., Halasa T., Toft N. (2015). Latent class analysis of bulk tank milk PCR and ELISA testing for herd level diagnosis of *Mycoplasma bovis*. Prev. Vet. Med..

[30] Spergser J., Macher K., Kargl M., Lysnyansky I., Rosengarten R. (2013). Emergence, re-emergence, spread and host species crossing of *Mycoplasma bovis* in the Austrian Alps caused by a single endemic strain. Vet. Microbiol..

[31] Van Engelen E., Dijkman R., Holzhauer M., Junker K., Van Wuyckhuise L., Gonggrijp M. Typing of *Mycoplasma bovis* from arthritis outbreaks in dairy herds. Proceedings of the European Mycoplasma Meeting: Progress in Human and Animal Mycoplasmology.

[32] Aebi M., van den Borne B.H.P., Raemy A., Steiner A., Pilo P., Bodmer M. (2015). *Mycoplasma bovis* infections in Swiss dairy cattle: A clinical investigation. Acta Vet. Scand..

[33] Arcangioli M.A., Chazel M., Sellal E., Botrel M.A., Bezille P., Poumarat F., Calavas D., Le Grand D. (2011). Prevalence of *Mycoplasma bovis* udder infection in dairy cattle: Preliminary field investigation in southeast France. N. Z. Vet. J..

[34] Filioussis G., Christodoulopoulos G., Thatcher A., Petridou V., Bourtzi-Chatzopoulou E. (2007). Isolation of *Mycoplasma bovis* from bovine clinical mastitis cases in Northern Greece. Vet. J..

[35] Nicholas R.A.J., Fox L.K., Lysnyansky I. (2016). Mycoplasma mastitis in cattle: To cull or not to cull. Vet. J..

[36] Passchyn P., Piepers S., De Meulemeester L., Boyen F., Haesebrouck F., De Vliegher S. (2012). Between-herd prevalence of *Mycoplasma bovis* in bulk milk in Flanders, Belgium. Res. Vet. Sci..

[37] Klein U., de Jong A., Moyaert H., El Garch F., Leon R., Richard-Mazet A., Rose M., Maes D., Pridmore A., Thomson J.R. (2017). Antimicrobial susceptibility monitoring of Mycoplasma hyopneumoniae and *Mycoplasma bovis* isolated in Europe. Vet. Microbiol..

[38] Klein U., de Jong A., Youala M., El Garch F., Stevenin C., Moyaert H., Rose M., Catania S., Gyuranecz M., Pridmore A. (2019). New antimicrobial susceptibility data from monitoring of *Mycoplasma bovis* isolated in Europe. Vet. Microbiol..

[39] Ministerio de Agricultura, Pesca y Alimentación. https://www.mapa.gob.es/.

[40] Haapala V., Pohjanvirta T., Vähänikkilä N., Halkilahti J., Simonen H., Pelkonen S., Soveri T., Simojoki H., Autio T. (2018). Semen as a source of *Mycoplasma bovis* mastitis in dairy herds. Vet. Microbiol..

[41] Becker C.A.M., Thibault F.M., Arcangioli M.-A., Tardy F. (2015). Loss of diversity within *Mycoplasma bovis* isolates collected in France from bovines with respiratory diseases over the last 35 years. Infect. Genet. Evol..

[42] Khalil D., Becker C.A.M., Tardy F. (2016). Alterations in the quinolone resistance-determining regions and fluoroquinolone resistance in clinical isolates and laboratory-derived Mutants of *Mycoplasma bovis*: Not all genotypes may be equal. Appl. Environ. Microbiol..

[43] Khalil D., Becker C.A.M., Tardy F. (2017). Monitoring the decrease in susceptibility to ribosomal RNAs targeting antimicrobials and its molecular basis in clinical *Mycoplasma bovis* isolates over time. Microb. Drug Resist. Larchmt. N.

[44] Lysnyansky I., Mikula I., Gerchman I., Levisohn S. (2009). Rapid detection of a point mutation in the parC gene associated with decreased susceptibility to fluoroquinolones in *Mycoplasma bovis*. Antimicrob. Agents Chemother..

[45] Mustafa R., Qi J., Ba X., Chen Y., Hu C., Liu X., Tu L., Peng Q., Chen H., Guo A. (2013). In vitro quinolones susceptibility analysis of Chinese *Mycoplasma bovis* isolates and their phylogenetic scenarios based upon QRDRs of DNA topoisomerases revealing a unique transition in ParC. Pak. Vet. J..

[46] Sato T., Okubo T., Usui M., Higuchi H., Tamura Y. (2013). Amino acid substitutions in GyrA and ParC are associated with fluoroquinolone resistance in *Mycoplasma bovis* isolates from Japanese dairy calves. J. Vet. Med. Sci..

[47] Sulyok K.M., Kreizinger Z., Wehmann E., Lysnyansky I., Bányai K., Marton S., Jerzsele Á., Rónai Z., Turcsányi I., Makrai L. (2017). Mutations associated with decreased susceptibility to seven antimicrobial families in field and laboratory-derived *Mycoplasma bovis* strains. Antimicrob. Agents Chemother..

[48] Hata E., Harada T., Itoh M. (2019). Relationship between antimicrobial susceptibility and multilocus sequence type of *Mycoplasma bovis* isolates and development of a method for rapid detection of point mutations involved in decreased susceptibility to macrolides, lincosamides, tetracyclines, and spectinomycin. Appl. Environ. Microbiol..

[49] Lerner U., Amram E., Ayling R.D., Mikula I., Gerchman I., Harrus S., Teff D., Yogev D., Lysnyansky I. (2014). Acquired resistance to the 16-membered macrolides tylosin and tilmicosin by *Mycoplasma bovis*. Vet. Microbiol..

[50] Lysnyansky I., Gerchman I., Flaminio B., Catania S. (2015). Decreased susceptibility to macrolide-lincosamide in mycoplasma synoviae is associated with mutations in 23S ribosomal RNA. Microb. Drug Resist..

[51] Amram E., Mikula I., Schnee C., Ayling R.D., Nicholas R.A.J., Rosales R.S., Harrus S., Lysnyansky I. (2015). 16S rRNA gene mutations associated with decreased susceptibility to tetracycline in *Mycoplasma bovis*. Antimicrob. Agents Chemother..

[52] CIMAVet Centro de Información de Medicamentos para Veterinaria. https://cimavet.aemps.es/.

[53] Stipkovits L., Ripley P.H., Tenk M., Glávits R., Molnár T., Fodor L. (2005). The efficacy of valnemulin (Econor) in the control of disease caused by experimental infection of calves with *Mycoplasma bovis*. Res. Vet. Sci..

[54] Waites K.B., Bébéar C.M., Robertson J.A., Talkington D.F., Kenny G.E. (2001). Cumitech 34: Laboratory Diagnosis of Mycoplasmal Infections.

[55] Tola S., Angioi A., Rocchigiani A.M., Idini G., Manunta D., Galleri G., Leori G. (1997). Detection of *Mycoplasma agalactiae* in sheep milk samples by polymerase chain reaction. Vet. Microbiol..

[56] Foddai A., Idini G., Fusco M., Rosa N., De la Fe C., Zinellu S., Corona L., Tola S. (2005). Rapid differential diagnosis of *Mycoplasma agalactiae* and *Mycoplasma bovis* based on a multiplex-PCR and a PCR-RFLP. Mol. Cell. Probes.

[57] Tamura K., Stecher G., Peterson D., Filipski A., Kumar S. (2013). MEGA6: Molecular evolutionary genetics analysis version 6.0. Mol. Biol. Evol..

[58] Gómez-Martín A., De la Fe C., Amores J., Sánchez A., Contreras A., Paterna A., Buendía A.J., Corrales J.C. (2012). Anatomic location of *Mycoplasma mycoides* subsp. *capri* and *Mycoplasma agalactiae* in naturally infected goat male auricular carriers. Vet. Microbiol..

[59] Albers A.C., Fletcher R.D. (1982). Simple method for quantitation of viable mycoplasmas. Appl. Environ. Microbiol..

[60] Epi Info^TM^|CDC. https://www.cdc.gov/epiinfo/index.html.

[61] Babraham Bioinformatics—FastQC A Quality Control Tool for High Throughput Sequence Data. https://www.bioinformatics.babraham.ac.uk/projects/fastqc/.

[62] Li H., Durbin R. (2009). Fast and accurate short read alignment with Burrows-Wheeler transform. Bioinformatics.

[63] Okonechnikov K., Conesa A., García-Alcalde F. (2016). Qualimap 2: Advanced multi-sample quality control for high-throughput sequencing data. Bioinformatics.

[64] Thorvaldsdóttir H., Robinson J.T., Mesirov J.P. (2013). Integrative Genomics Viewer (IGV): High-performance genomics data visualization and exploration. Brief. Bioinform..

[65] Deatherage D.E., Barrick J.E. (2014). Identification of mutations in laboratory-evolved microbes from next-generation sequencing data using breseq. Engineering and Analyzing Multicellular Systems.

