# Supplementary material for: Mycoplasma bovis in Spanish Cattle Herds: Two Groups of Multiresistant Isolates Predominate, with One Remaining Susceptible to Fluoroquinolones
Source: Pathogens. 2020 Jul 7;9(7):545. doi: 10.3390/pathogens9070545 (PMC7399988; doi:10.3390/pathogens9070545)
Supplement: Supplementary file 1 [file pathogens-09-00545-s001.zip › Figure S1. Map of Spain showing the autonomous communities (AC) and the origin of the samples.docx]

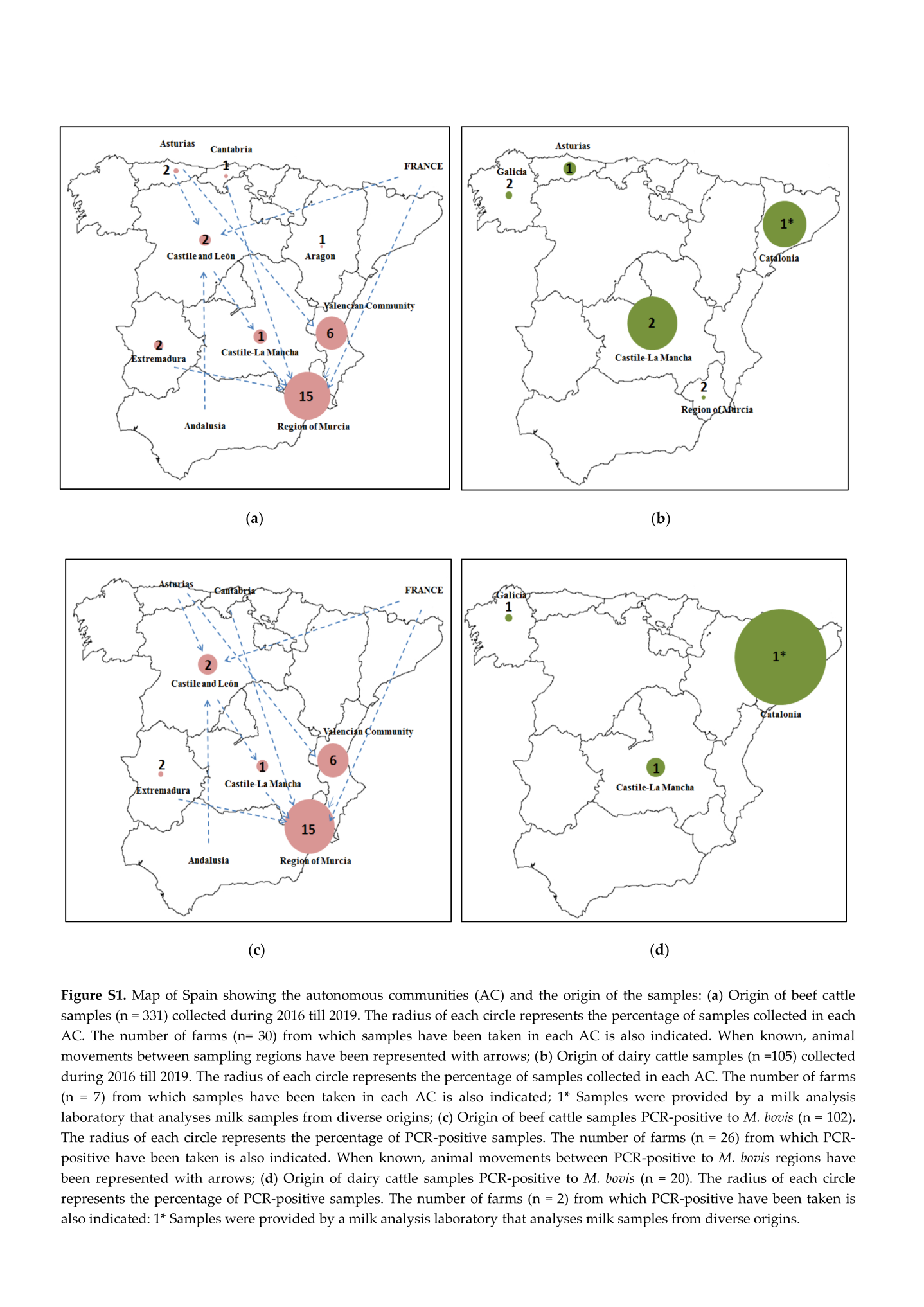


**Figure S1.** Map of Spain showing the autonomous communities (AC) and the origin of the samples: (**a**) Origin of beef cattle samples (n = 331) collected during 2016 till 2019. The radius of each circle represents the percentage of samples collected in each AC. The number of farms (n = 30) from which samples have been taken in each AC is also indicated. When known, animal movements between sampling regions have been represented with arrows; (**b**) Origin of dairy cattle samples (n = 105) collected during 2016 till 2019. The radius of each circle represents the percentage of samples collected in each AC. The number of farms (n = 7) from which samples have been taken in each AC is also indicated; 1* Samples were provided by a milk analysis laboratory that analyses milk samples from diverse origins; (**c**) Origin of beef samples PCR-positive to *M. bovis*  (n = 102). The radius of each circle represents the percentage of PCR-positive samples. The number of farms (n = 26) from which PCR-positive have been taken is also indicated. When known, animal movements between PCR-positive to *M. bovis* regions have been represented with arrows; (**d**) Origin of dairy cattle samples PCR-positive to *M. bovis* (n = 20). The radius of each circle represents the percentage of PCR-positive samples. The number of farms (n = 2) from which PCR-positive have been taken is also indicated: 1* Samples were provided by a milk analysis laboratory that analyses milk samples from diverse origins.
