# Supplementary material for: Mycoplasma bovis in Spanish Cattle Herds: Two Groups of Multiresistant Isolates Predominate, with One Remaining Susceptible to Fluoroquinolones
Source: Pathogens. 2020 Jul 7;9(7):545. doi: 10.3390/pathogens9070545 (PMC7399988; doi:10.3390/pathogens9070545)
Supplement: Supplementary file 1 [file pathogens-09-00545-s001.zip › Table S2. Partial sequences (520pb) types of the polC gene.docx]

**Table S2.** Partial sequences (520pb) types of the *polC* gene.

| > Subtype 2 |
| --- |
| CAAATTATTTAATAAAGCAAAAAAATCAGGAATTAAAGCCATATACGGAACTGCATTCACTACTATAAATAAGGCAAATGAGGCAATTTTAGGCGAAATTCCTGAGGGCAGTTTTAAAGACTATTCATATGTTTCATTTGACATTGAAACAACAGGATTAAGTCCAAAATTCCACGAAATAATTGAATTTGGTGCAGTTGATATTAATCAGTATTTAAAAGTAGGAAAAACAACTCAGTTTTTTATTAAGCCAAAAGATAAAATTGGTAGTTTTACTACTGAATTAACCGGAATTACACAGCAAATGCTTGATTCTAAGGGGCTTTATATAAAAGAAGGTCTAGAAAAAATTTATGATTGTTTAGATGGAAAAATAGCTATTGCACATAATGCAAAATTTGACTTTAACTTTTTAAAGGAACAATTTAGGCTCAACAATATGCAATTCCCAAGAGTCACAGTTATTGACACTCTGGTAGCTTCTAGAATTGGTTTCCCGGGCTATAAACGCCACAAATTA |
| > Subtype 3 |
| CAAATTATTTAATAAAGCAAAAAAATCAGGAATTAAAGCCATATACGGAACTGCATTCACTACTATAAATAAGGCAAATGAGGCAATTTTAGGCGAAATTCCTGAAGGCAGTTTTAAAGACTATCCATATGTTTCATTTGACATTGAAACAACAGGATTAAGTCCAAAATTCCACGAAATAATTGAATTTGGTGCAGTTGATATTAATCAAGATTTAAAGGTGGGTAAAACAACTCAGTTTTTTATCAAGCCAAAAGATAAAATTGGTAGTTTTACTACTGAATTAACTGGAATTACACAGCAAATGCTTGATTCTAAGGGGCTTGATATAAAAGAAGGTCTAGAAAAAATTTATGATTGTTTAGACGGAAAAATAGCTATTGCACATAATGCAAAATTTGACTTTAACTTTTTAAAGGAACAATTTAGACTCAACAATATGCAATTCCCAAGAGTCACTGTTATTGACACTCTGGTAGCTTCTAGAATTGGCTTTCCGGGATATAAACGTCACAAATTA |
